# The HIF-1A/miR-17-5p/PDCD4 axis contributes to the tumor growth and metastasis of gastric cancer

**DOI:** 10.1038/s41392-020-0132-z

**Published:** 2020-04-10

**Authors:** Jiayu Zhao, Anqi Xiao, Chunyu Liu, Chao Ye, Kai Yin, Minghon Lu, Ruihua Jiao, Xi Chen, Chenyu Zhang, Minghui Liu

**Affiliations:** 1 School of Life Sciences, Nanjing University, Nanjing, Jiangsu 210046 China; 2 School of Life Sciences, China Pharmaceutical University, Nanjing, Jiangsu China; 3 Ma’anshan Municipal Health Commission, Ma’anshan, Anhui 22540 China; 4 Taixing Hospital Affiliated to Kangda college, Nanjing Medical University, Taixing, Jiangsu 225400 China; 5 University College London, Bloomsbury, London, WC1E 6BT United Kingdom

**Keywords:** Non-coding RNAs, Gastrointestinal cancer

**Dear Editor,**

Gastric cancer (GC) is one of the most common malignant tumors of the digestive system and the second leading cause of cancer death worldwide^1^. Despite the gradually declining morbidity and mortality, GC still burdens many countries in East Asia^1^. Understanding the GC pathological process is vital for the successful diagnosis, treatment and prevention of this disease.

Programmed cell death 4 (PDCD4), a nucleocytoplasmic shuttling protein, binds to and exerts an inhibitory effect on the helicase activity of eukaryotic translation initiation factor 4A (EIF4A), which is an RNA helicase catalyzing the unwinding of mRNA secondary structure^2^. By combining these two mechanisms, PDCD4 suppresses the translation of specific mRNAs. PDCD4 has become a hotspot of cancer research as a newly identified tumor suppressor gene in recent years^3^. The roles of PDCD4 in GC mainly include the inhibition of cell proliferation and metastasis and the promotion of cell apoptosis^4,5^. Despite a growing number of investigations, the underlying mechanism of PDCD4 in GC remains to be fully clarified.

microRNAs (miRNAs) play an extensive role in various physiological and pathological processes. These single-stranded noncoding RNAs of 18–25 nucleotides can block mRNA translation by targeting the 3’ untranslated region (3’UTR)^6^. In a previous study by our lab^7^, ten miRNAs, including miR-17-5p, were identified as the most highly upregulated miRNAs in gastric tumor tissues. Among these miRNAs, miR-16-5p, miR-23b-3p, let-7a-5p, miR-15a-5p, miR-17-5p and miR-93 were identified as candidate regulators of PDCD4, indicating that PDCD4 could be the key downstream protein during GC development. Specifically, miR-17-5p and miR-93 were shown to most likely regulate PDCD4. However, it remains unclear whether miR-17-5p could regulate PDCD4 during GC development.

In this study, we found that decreased PDCD4 mRNA expression was negatively correlated with increased miR-17-5p levels in GC tumor tissues according to TCGA datasets (Supplementary Fig. S1a–c). This negative relationship between PDCD4 and miR-17-5p levels was also confirmed in 16 pairs of GC tumor tissues and their adjacent normal tissues (Supplementary Fig. S1d–i). Both low PDCD4 expression and high miR-17-5p levels led to worse overall survival (OS) and recurrence-free survival (RFS) outcomes for GC patients according to the TCGA dataset (Supplementary Fig. S1j).

Mechanistic investigation indicated a potential regulatory site of miR-17-5p in the PDCD4 3’UTR (Fig. 1a). After overexpressing or silencing miR-17-5p using transfection of pre-miR-17-5p or anti-miR-17-5p (Supplementary Fig. S2a), we determined that miR-17-5p could inhibit PDCD4 expression (Fig. 1b, Supplementary Fig. S2b, c). Transfection of the PDCD4 plasmid rescued the miR-17-5p-silenced PDCD4 levels (Supplementary Fig. S2d–h). The luciferase reporter assay demonstrated direct binding between miR-17-5p and the PDCD4 3′UTR (Fig. 1c). We also demonstrated that miR-17-5p promoted MKN-45 cell proliferation and migration and prevented MKN-45 apoptosis by suppressing PDCD4 (Supplementary Fig. S3). In the orthotopic mouse model, PDCD4 effectively inhibited tumor growth and liver metastasis, whereas miR-17-5p resulted in faster tumor growth and worse liver metastasis. PDCD4 overexpression restored the cancer-promoting effect of miR-17-5p (Fig. 1d). Moreover, the xenograft mouse model proved that miR-17-5p plays an oncogenic role by repressing PDCD4 expression (Supplementary Fig. S4).

**Fig. 1:**
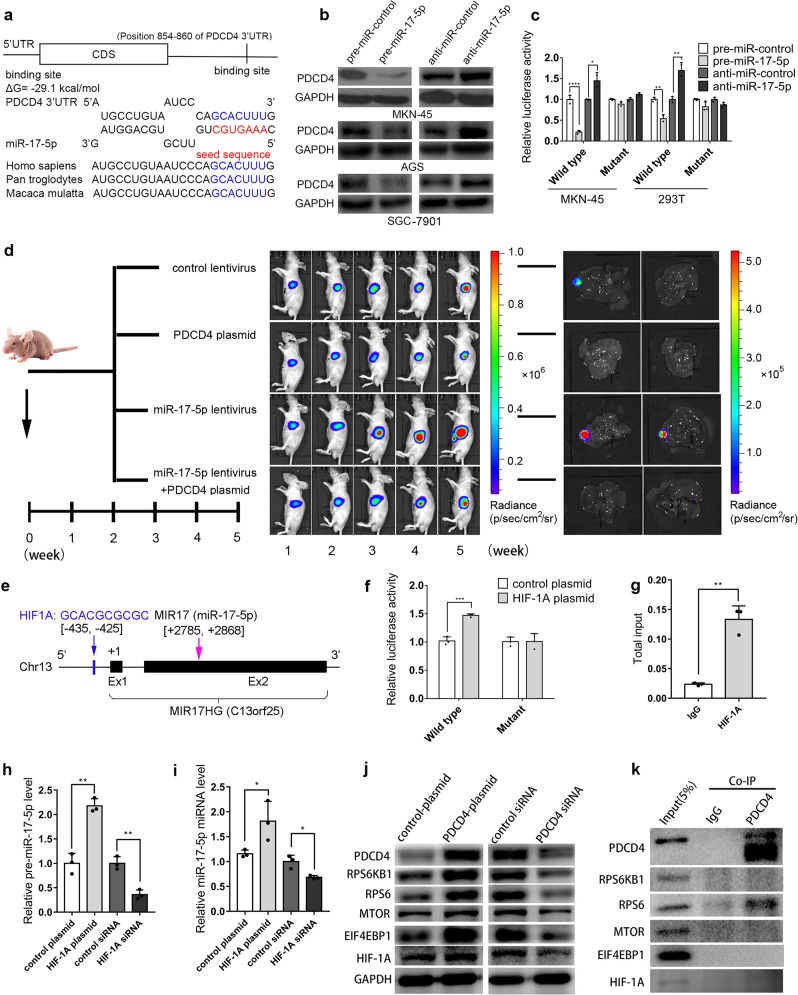
**a** Potential binding site of miR-17-5p at the PDCD4 3′UTR. **b** PDCD4 was negatively regulated by miR-17-5p in GC cells. **c** Relative luciferase activities in MKN-45 and 293T cells treated with pre-miR-17-5p or anti-miR-17-5p. **d** miR-17-5p promotes GC tumor growth and metastasis in vivo by targeting PDCD4. **e** Potential targeting of HIF-1A at the promoter region of miR-17-5p. **f**, **g** Luciferase reporter and ChIP assays: HIF-1A targets the promoter region of miR-17-5p. **h**, **i** HIF-1A increases the levels of pre-miR-17-5p and miR-17-5p. **j** PDCD4 enhances the expression of RPS6KB1, RPS6, MTOR, EIF4EBP1 and HIF-1A. **k** Co-IP assay: RPS6 binds with PDCD4. *P < 0.05; **P < 0.01; ***P < 0.001; ****P < 0.0001

To date, less is known about why miR-17-5p is overexpressed during GC progression. In this study, we identified 4 transcription factors that could potentially target the miR-17-5p promoter region (Supplementary Fig. S5a). The Oncomine dataset showed that TFAP2A (transcription factor AP-2 alpha) and HIF-1A (hypoxia inducible factor 1 subunit alpha) were overexpressed in GC tumors (Supplementary Fig. S5b–e). Bioinformatic analysis indicated that overexpression of TFAP2A or HIF-1A was negatively associated with the survival outcomes of GC patients (Supplementary Fig. S5f) and positively correlated with miR-17-5p levels in GC tumors (Supplementary Fig. S5g, h). However, we found that HIF-1A was highly expressed (Supplementary Fig. S6a–c) in the tumor tissue of 16 GC tissue pairs, while TFAP2A was decreased (Supplementary Fig. S6e, f). Given that only HIF-1A levels in GC tumor tissues were consistent with the previously predicted results, we focused on HIF-1A in our further study. The subsequent results showed that HIF-1A could potentially target the promoter region of miR-17-5p (Fig. 1e). By overexpressing or silencing HIF-1A (Supplementary Fig. S2j–l), we confirmed the direct target of HIF-1A to the miR-17-5p promoter region using luciferase reporter and ChIP assays (Fig. 1f, g). Consequently, HIF-1A activated the transcription of pre-miR-17-5p and miR-17-5p (Fig. 1h, i, Supplementary Figs. S1g, S6d, g, h). Our results suggest that HIF-1A-activated miR-17-5p overexpression promotes GC development and metastasis by repressing PDCD4.

When analyzing the data of cell transfections and the xenograft mouse model, we found that PDCD4 overexpression resulted in low miR-17-5p levels (Supplementary Figs. S2i and S4c), the underlying mechanism of which deserves special attention and investigation. We performed PPI, GO and KEGG enrichment analyses to figure out a PPI network of PDCD4 involving genes such as the EIF family, STAT4, RPS6KB1 and mTOR (Supplementary Fig. S7a–c). Interestingly, the KEGG analysis revealed that PDCD4 might participate in the HIF-1 signaling pathway through potential interactions with EIF4EBP1, MTOR, RPS6 and RPS6KB1 (Supplementary Fig. S7d). By further experiments, we found that PDCD4 could positively regulate the expression of HIF-1A, EIF4EBP1, MTOR, RPS6 and RPS6KB1 (Fig. 1j). In particular, PDCD4 could directly bind to RPS6 (Fig. 1k). This was an unexpected finding. The mTOR/RPS6KB1/RPS6/EIF4EBP1 signaling pathway is well known to enhance HIF-1A transcription levels^8^. Our result might imply that PDCD4 enhances HIF-1A expression by directly activating RPS6. However, it remains unclear how PDCD4 could negatively regulate the miR-17-5p level in gastric tumors, which deserves more attention and further investigation in future study.

In conclusion, we demonstrated that the HIF-1A/miR-17-5p/PDCD4 axis contributes to the carcinogenesis of gastric cancer. Furthermore, we determined that PDCD4 could enhance the HIF-1A signaling pathway and thereby form a negative feedback loop of PDCD4/HIF-1A/miR-17-5p/PDCD4. We inferred that the biological function of this regulation might be a self-rescue system for the decrease in HIF-1A expression through PDCD4 downregulation in the tumorigenesis of gastric cancer. Our study provides new insights into gastric tumor etiology and potential targets for GC treatment.

## Supplementary information

Supplemental information
