# Supplementary material for: The HIF-1A/miR-17-5p/PDCD4 axis contributes to the tumor growth and metastasis of gastric cancer
Source: Signal Transduct Target Ther. 2020 Apr 10;5:46. doi: 10.1038/s41392-020-0132-z (PMC7145839; doi:10.1038/s41392-020-0132-z)
Supplement: Supplementary file 1 — Supplemental information [file 41392_2020_132_MOESM1_ESM.docx]

Supplementary Materials for

The HIF-1A/miR-17-5p/PDCD4 axis contributes to the tumor growth and metastasis of gastric cancer

Jiayu Zhao^1*^, Anqi Xiao^1*^, Chunyu Liu^1*^, Chao Ye^3^, Kai Yin^4^, Minghon Lu^5^, Ruihua Jiao^1+^, Xi Chen^1+^, Chenyu Zhang^1+^, Minghui Liu^1, 2+^.

1. State Key Laboratory of Pharmaceutical Biotechnology, Collaborative Innovation Center of Chemistry for Life Sciences, Jiangsu Engineering Research Center for MicroRNA Biology and Biotechnology, NJU Advanced Institute for Life Sciences (NAILS), School of Life Sciences, Nanjing University, Nanjing, Jiangsu 210046, China;
2. School of Life Sciences, China Pharmaceutical University, Nanjing, Jiangsu, China;
3. Ma'anshan Municipal Health Commission, No. 360 Yingcui Road, Ma'anshan, Anhui 243000, China;
4. Department of General Surgery, Taixing Hospital Affiliated to Kangda college, Nanjing Medical University, 1 Changzheng Road, Taixing, Jiangsu 225400, China.
5. Department of Biochemical Engineering, Faculty of Chemical Engineering, University College London, Gower Street, Bloomsbury, London, WC1E6BT, United Kingdom

*These authors contributed equally to this work.

Correspondence to: Minghui Liu (minghuiliu@nju.edu.cn), Chenyu Zhang (cyzhang@nju.edu.cn), Xi Chen (xichen@nju.edu.cn) and Ruihua Jiao (rhjiao@nju.edu.cn)

**This PDF file includes:**

Materials and Methods

Supplementary Text

Figures. S1 to S7

Tables S1 to S2

Materials and Methods

**Cells and tissues**

GC tissues and normal adjacent tissues were acquired from the Affiliated Drum Tower Hospital of Nanjing University Medical School (Nanjing, China). All patients signed the consent agreement and every aspect of this study was approved by the Ethics Committee of Nanjing University. **Supplemental Table 1** listed the clinical characteristics of GC patients. Human gastric cancer cell lines MKN-45, AGS and SGC were purchased from the Shanghai Institute of Cell Biology, Chinese Academy of Sciences (Shanghai, China). All cells were cultured in RPMI 1640 medium (Invitrogen, USA) supplemented with 10% fetal bovine serum (FBS, Gibco, USA) in a 5% CO2 water-saturated atmosphere at 37 °C.

**RNA isolation and quantitative RT-PCR (qRT-PCR)**

Total RNA was extracted with Trizol reagent (Sigma, USA). NanoDrop (Thermo, USA) were used to detect RNA concentrations. miRNA quantifications were conducted with TaqMan miRNA probes (Applied Biosystems, USA) while mRNAs were quantified with SYBR Green dye (Ambion, USA) and specific primers. 1 μg of total RNA was reverse-transcribed to cDNA by AMV reverse transcriptase (TaKaRa, China). After qRT-PCR reactions, the cycle threshold (CT) data were determined using fixed threshold settings and the mean CT was calculated from triplicate reaction wells. U6 snRNA or GAPDH was used as an internal control (IC). The relative levels of miRNA or mRNA normalized to the internal control were calculated using the equation 2^–△△CT^, in which △△CT= (CT _miR-17-5p or mRNA_ – CT _U6 or GAPDH_) _tumor_ – (CT _miR-17-5p or mRNA_ – CT _U6 or GAPDH_) _control_. All sequences were listed in **Supplemental Table 2.**

**Protein extraction and western blot (WB)**

RIPA lysis buffer (Beyotime, Shanghai, China) was used to extract total protein when supplemented with PMSF (Beyotime, Shanghai, China). The protein concentration was quantified by BCA protein assay kit (Thermo Scientific, USA). SDS-PAGE (Bio-Rad) was used to separate proteins. GAPDH was used as the internal control. Antibodies against PDCD4 (sc-130545, 1:1000), HIF1A (ab16066, 1:1000), RPS6KB1 (ab32529, 1:1000), RPS6 (ab225676, 1:1000), mTOR (ab2732, 1:1000), EIF4EBP1 (ab32024, 1:1000), TFAP2A (ab52222, 1:1000) and GAPDH (sc-25778, 1:1000) were purchased from Santa Cruz Biotechnology, USA.

**miR-17-5p overexpression and knockdown**

miR-17-5p overexpression and knockdown were achieved by transfecting GC cells with pre-miR-17-5p (RiboBio, Guangzhou, China) and anti-miR-17-5p (RiboBio, Guangzhou, China), respectively. Lipofectamine 2000 (Invitrogen, USA) was used to help transfection. Cell culture medium was changed to RPMI-1640 supplemented with 2% FBS at 6 h after transfection. The transfected cells were cultured until further experiments.

**SiRNA interference and plasmid construction**

A mammalian expression plasmid (PDCD4 plasmid) was designed to express the full-length open reading frame (ORF) of the human PDCD4 gene (Genescript, Nanjing, China). An empty plasmid served as a negative control (control plasmid). Synthetic siRNA (PDCD4 siRNA) was designed to silence PDCD4 (GenePharma, Shanghai, China). A scrambled siRNA served as a control. PDCD4 plasmid and PDCD4 siRNA were transfected into GC cells with Lipofectamine 2000. Transfected cells were cultured until further experiments. Similarly, HIF-1A was silenced or overexpressed by transfection of HIF-1A siRNA or HIF-1A plasmid.

**Luciferase reporter assay**

pMIR-report luciferase vectors were purchased from Ambion (Carlsbad, CA, USA). A fragment of PDCD4 3’UTR containing the miR-17-5p binding sites was inserted into pMIR-report vectors to construct a wild type luciferase plasmid. The miR-17-5p binding site sequence was mutated from GCACTTT to CGTGAAA in order to construct a mutant luciferase plasmid. For luciferase reporter assays, MKN-45 and 293T cells were co-transfected with a luciferase reporter plasmid, a β-galactosidase (β-gal) expression plasmid (Ambion, USA), pre-miR-17-5p and anti-miR-17-5p. The β-gal plasmid was used as a transfection efficiency control. Luciferase activity was tested by a luciferase assay kit (Promega Madison, WI, USA).

**Cell apoptosis assay**

MKN-45 cells were transfected with the above-mentioned RNAs or plasmids. After transfection, LPS (Sigma) was added with a final concentration of 400 ng/mL to induce apoptosis. Cells were harvested after culturing for 24 h in FBS-depleted medium. Cell apoptosis was assayed by flow cytometric analysis with an Annexin V-FITC/PI staining kit (BD Biosciences, USA) according to the manufacturer’s instruction.

**Cell proliferation assay**

MKN-45 cells were transfected with the above-mentioned RNAs or plasmids and then reseeded in 96-well plates. The cell proliferation index was measured using the Cell Counting Kit-8 (CK04-500, Dojindo, Japan) at 12, 24, 36, 48, 60 and 72 h post-transfection according to the manufacturer’s instruction. Absorbance was measured at a wavelength of 450 nm.

**Transwell migration assay**

Cell migration assay was performed using Millipore 24-Well Millicell (Millipore) plates containing an 8-μm pore membrane. The bottom face of the membrane was coated with 1% gelatin. RPMI-1640 supplemented with 10% FBS was added to the lower compartment. Cells were suspended in FBS-free RPMI-1640 culture medium after transfection and reseeded into the upper chamber. After incubation for 48 h, cells were fixed with 4% paraformaldehyde for 25 min. The membrane was then stained with 0.1% crystal violet in methanol for 15 min. After washing, cells remaining on the upper surface (non-migrant) were gently scraped off with a cotton swab. The lower surfaces with migrant cells were captured and blindly counted.

**Cell wound-healing assay**

MKN-45 cells were transfected with the above-mentioned RNAs or plasmids and then reseeded in 12-well plates. After the cells have filled the bottom of the well, draw a line in the center with tips and take a picture of the width of the line at 0h and 24h after the underline.

**Xenografted mice construction**

4-week-old male nude mice were purchased from the Model Animal Research Center of Nanjing University (Nanjing, China). The miR-17-5p genomic sequence was cloned into a lentiviral expression vector (miR-17-5p lentivirus). MKN-45 cells were infected with miR-17-5p lentivirus, PDCD4 plasmid, or co-transfected with a miR-17-5p lentivirus plus PDCD4 plasmid. After transfection, MKN45 cells were subcutaneously injected into the armpits of nude mice. Mice were sacrificed 30 days later. Tumors were removed for weight measurement, RNA and protein extraction Hematoxylin and Eosin (H&E) staining and immunohistochemical (IHC) staining of PDCD4 (sc-376430, 1:500) and Ki-67 (CST, #9027, 1:400). All experiments were approved by the Ethics Committee of Nanjing University and complied with the U.K. Animals (Scientific Procedures) Act (1986) and the guidelines of the National Institutes of Health guides. For the orthotopic implantations, 1×10^7^ MKN-45 cells in 25 µL of PBS:matrigel (2:1, v/v) were injected between the gastric serosa and muscularis of nude mice (5 mice/group). The IVIS™ bioimaging system was used to conduct the *in vivo* bioluminescence imaging every 7 days with VivoGlo™ Luciferin (Promega) as a substrate. Mice were euthanized 35 days after implantation, and liver metastatic lesions were analysed using the IVIS™ bioimaging system.

**Chromatin immunoprecipitation (ChIP) assay**

A commercial kit (Beyotime, Shanghai, China) was used to perform CHIP assay according to the manufacturer’s instruction. HIF-1A antibody was used to immunoprecipitate specific HIF-1A-chromatin complexes. Anti-IgG (Santa Cruz, USA) served as a negative control. The ChIP products were amplified by qRT-PCR and then separated on 2% agarose gels. The primers for amplification are listed in **Supplemental Table** **2**.

**Co-Immunoprecipitation**

MKN-45 was transfected and collected after 48 hours. RIPA lysis buffer containing 1mM PMSF was used to fully lyse the cells at 4 °C. The supernatant was collected after centrifugation, 5% of which was isolated for protein extraction as the input group. The remaining section was added with IgG and PDCD4 antibody (1:500) respectively and incubated at 4 °C for 2h. Then the protein A / G plus agarose (GenScript, Nanjing, China) was added and shaken slowly at 4 °C overnight. The agarose beads were collected by centrifugation and rinsed for 3 times with PBS and then used for protein extraction as the IgG and PDCD4 group. WB was used to detect the obtained samples.

**Online database analysis**

The Cancer Genome Atlas (TCGA) database (https://www.cancer.gov/about-nci/organization/ccg/research/structural-genomics/tcga) and Oncomine database (https://www.oncomine.org/resource/login.html) were utilized to analyze the expression levels of miR-17-5p, PDCD4, TFAP2A, HIF-1A, NFIC and SRY in GC patients. Kaplan-Meier curves were used to analyze the survival rates of GC patients. To confirm PDCD4 is potentially targeted by miR-17-5p, Targetscan (http://www.targetscan.org/vert_72/) and RNAhybrid (https://bibiserv.cebitec.uni-bielefeld.de/rnahybrid) were used. To explore the upstream transcriptional factors regulating miR-17-5p, UCSC (http://genome.ucsc.edu/), PROMO (http://alggen.lsi.upc.es/cgi-bin/promo_v3/promo/promoinit.cgi?dirDB=TF_8.3) and JASPAR (http://jaspar.genereg.net/) were used. To predict the proteins interacting with PDCD4, STRING (https://string-db.org/) was utilized. Every database analysis was conducted according to the instructions and manuals.

**Statistical analysis**

All images are representatives of at least three independent experiments. The results are presented as the means ±SE of at least three independent experiments. Observed differences were considered statistically significant at P < 0.05 using Student’s t-test**.**

Supplementary Text

**Authors’ contributions**

Minghui Liu designed the study, acquired the funding and drafted the manuscript; Xi Chen and Chenyu Zhang financially supported this research; Ruihua Jiao helped to modify the manuscript; Jiayu Zhao, Anqi Xiao and Chunyu Liu carried out the main experiments; Chao Ye, Kai Yin and Minghon Lu provided material support.

**Ethics approval and consent to participate**

GC tissues were acquired from the Affiliated Drum Tower Hospital of Nanjing University Medical School (Nanjing, China). All patients signed the consent agreement and every aspect of this study was approved by the Ethics Committee of Nanjing University.

Figure. S1.


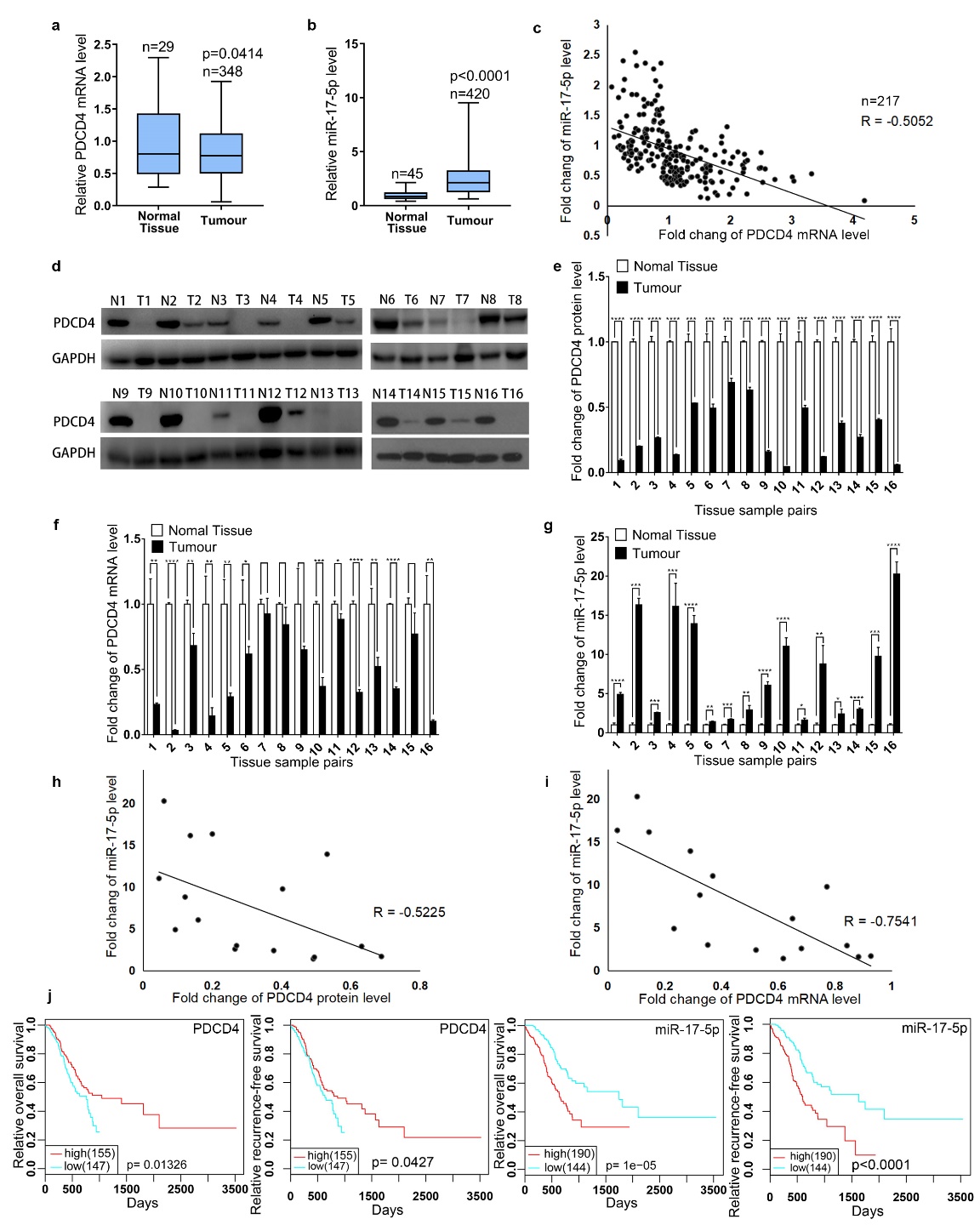


**Figure S1. PDCD4 downregulation is correlated with miR-17-5p overexpression in GC tissues, both of which lead to poor survival outcomes of GC patients.** (a and b) Analysis of PDCD4 and miR-17-5p expression levels in normal tissues and GC tumors in the TCGA dataset. (c) Pearson’s correlation coefficient between PDCD4 and miR-17-5p fold changes in GC patients from the TCGA dataset. (d-f) WB and qRT-PCR analysis: PDCD4 protein and mRNA levels in GC tissue pairs. (g) qRT-PCR analysis of miR-17-5p levels in GC tissue pairs. (h and i) Pearson’s correlation coefficient between miR-17-5p and PDCD4 protein as well as mRNA levels in GC tissue pairs. (j) Influence of PDCD4 and miR-17-5p expression on overall survival and recurrence free survival by Kaplan-Meier analysis in GC patients from TCGA dataset (cutoff value: 50% of PDCD4 mRNA or miR-17-5p expression). * P < 0.05; ** P < 0.01; *** P < 0.001; **** P<0.0001.

Figure. S2.


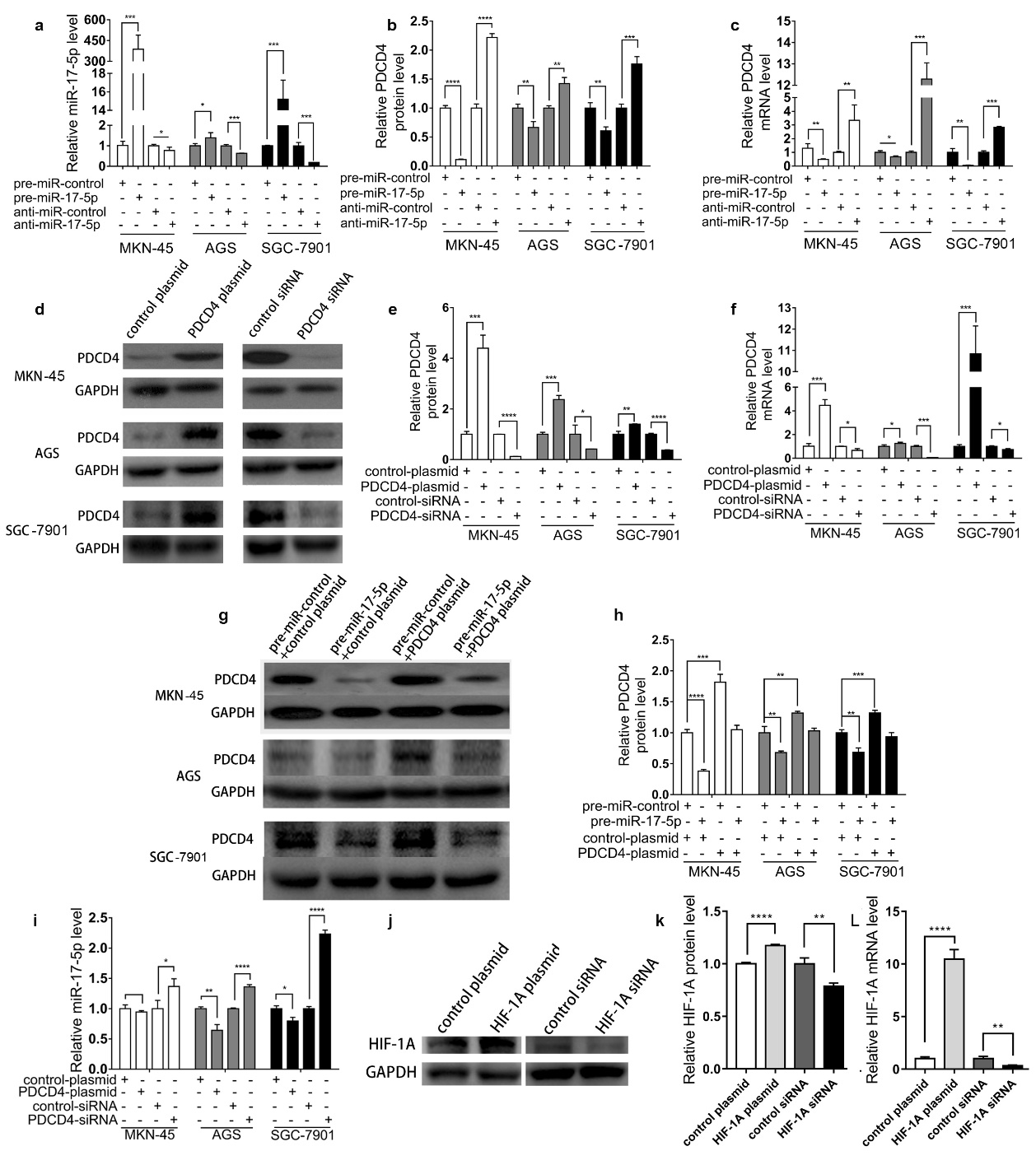


**Figure S2. Successful overexpression or knockdown of miR-17-5p, PDCD4 or HIF-1A.** (a) miR-17-5p was overexpressed or knocked down by pre-miR-17-5p or anti-miR-17-5p in MKN-45, AGS and SGC-7901 cells. (b and c) WB and qRT-PCR analysis: PDCD4 protein and mRNA levels were negatively affected by miR-17-5p in the three GC cells mentioned above. (d-f) WB and qRT-PCR analysis: PDCD4 was overexpressed or knocked down by PDCD4 plasmid or siRNA in three GC cells mentioned above. (g and h) WB analysis: PDCD4 plasmid could successfully restore the miR-17-5p-silenced PDCD4 levels. (i) miR-17-5p level was negatively affected by PDCD4 expressions in three GC cells mentioned above. (j-l) WB and qRT-PCR analysis: HIF-1A was overexpressed or knocked down by HIF-1A plasmid or siRNA in three GC cells mentioned above. * P < 0.05; ** P < 0.01; *** P < 0.001; **** P<0.0001.

Figure. S3.


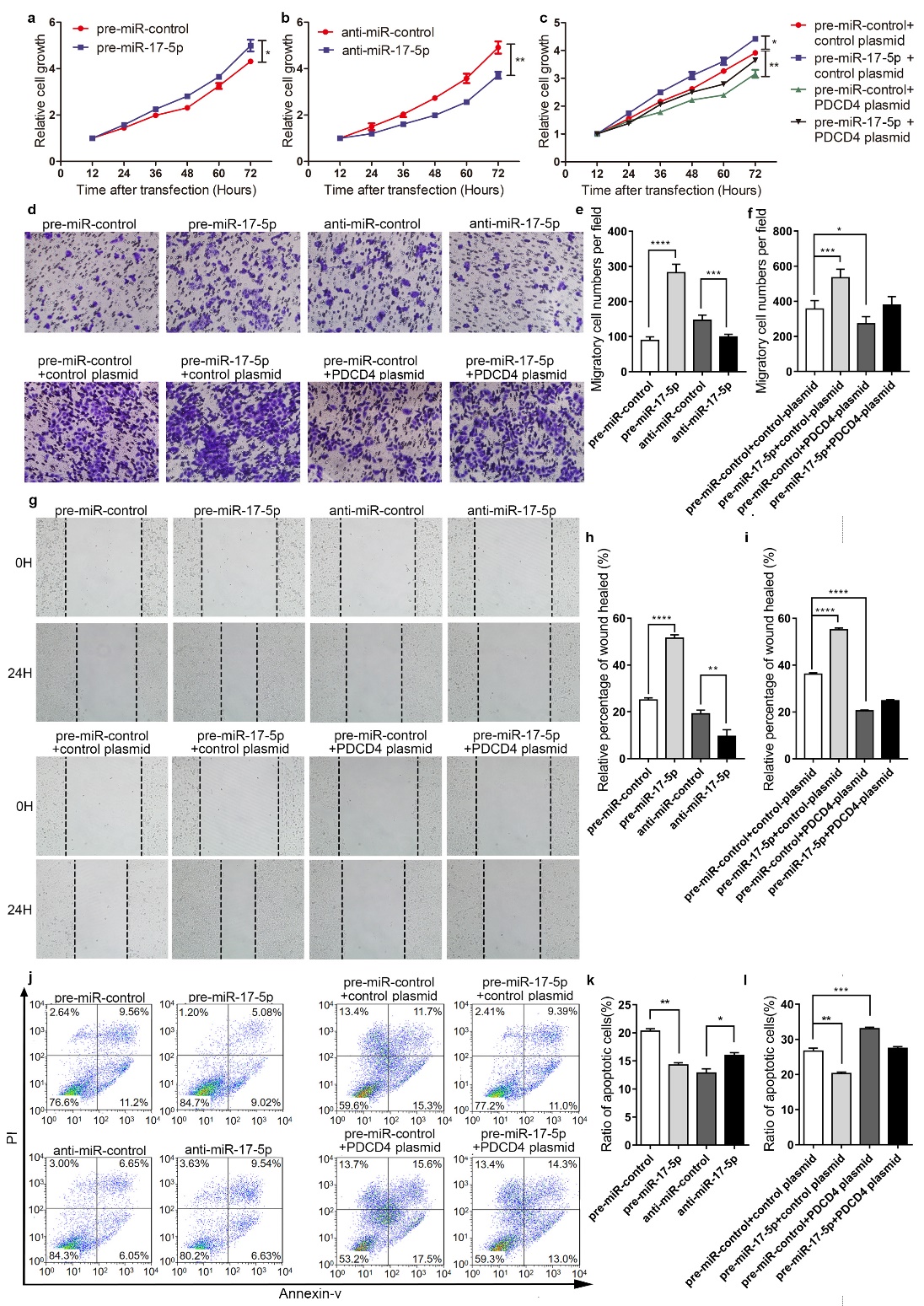


**Figure S3. miR-17-5p promotes MKN-45 proliferation and migration and inhibits MKN-45 apoptosis by suppressing PDCD4.** (a-b) CCK-8 assay: miR-17-5p promotes MKN-45 proliferation. (c) CCK-8 assay: miR-17-5p promotes MKN-45 proliferation by targeting PDCD4. (d-f) Migration assay and statistical analysis: miR-17-5p promotes MKN-45 migration by targeting PDCD4. (g-i) Wound-healing assay and statistical analysis: miR-17-5p promotes MKN-45 migration by targeting PDCD4. (j-l) Apoptosis assay and statistical analysis: miR-17-5p inhibits MKN-45 apoptosis by targeting PDCD4. * P < 0.05; ** P < 0.01; *** P < 0.001; **** P<0.0001.

Figure. S4.


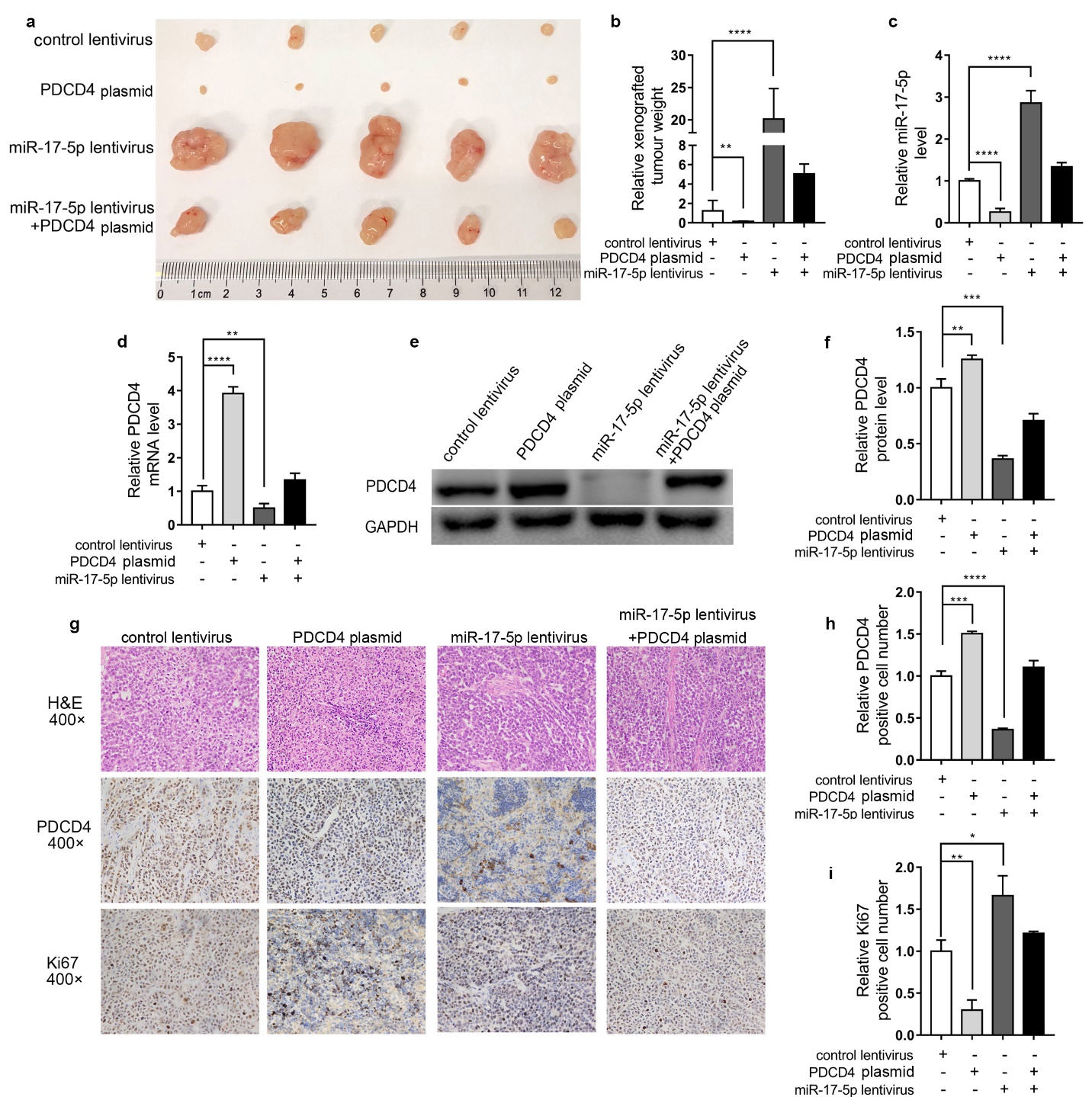


**Figure S4. miR-17-5p promotes GC tumor growth *in vivo* by targeting PDCD4.** (a and b) Photos of tumors and tumor weights: miR-17-5p accelerated but PDCD4 slowed down GC xenografted tumor growth. (c and d) qRT-PCR analysis of miR-17-5p and PDCD4 mRNA levels in GC xenografted tumors. (e and f) WB analysis of PDCD4 protein levels in GC xenografted tumors. (g-i) HE staining and IHC staining as well as statistical analysis for PDCD4 and Ki-67 in xenografted tumors. * P < 0.05; ** P < 0.01; *** P < 0.001; **** P<0.0001.

Figure. S5.


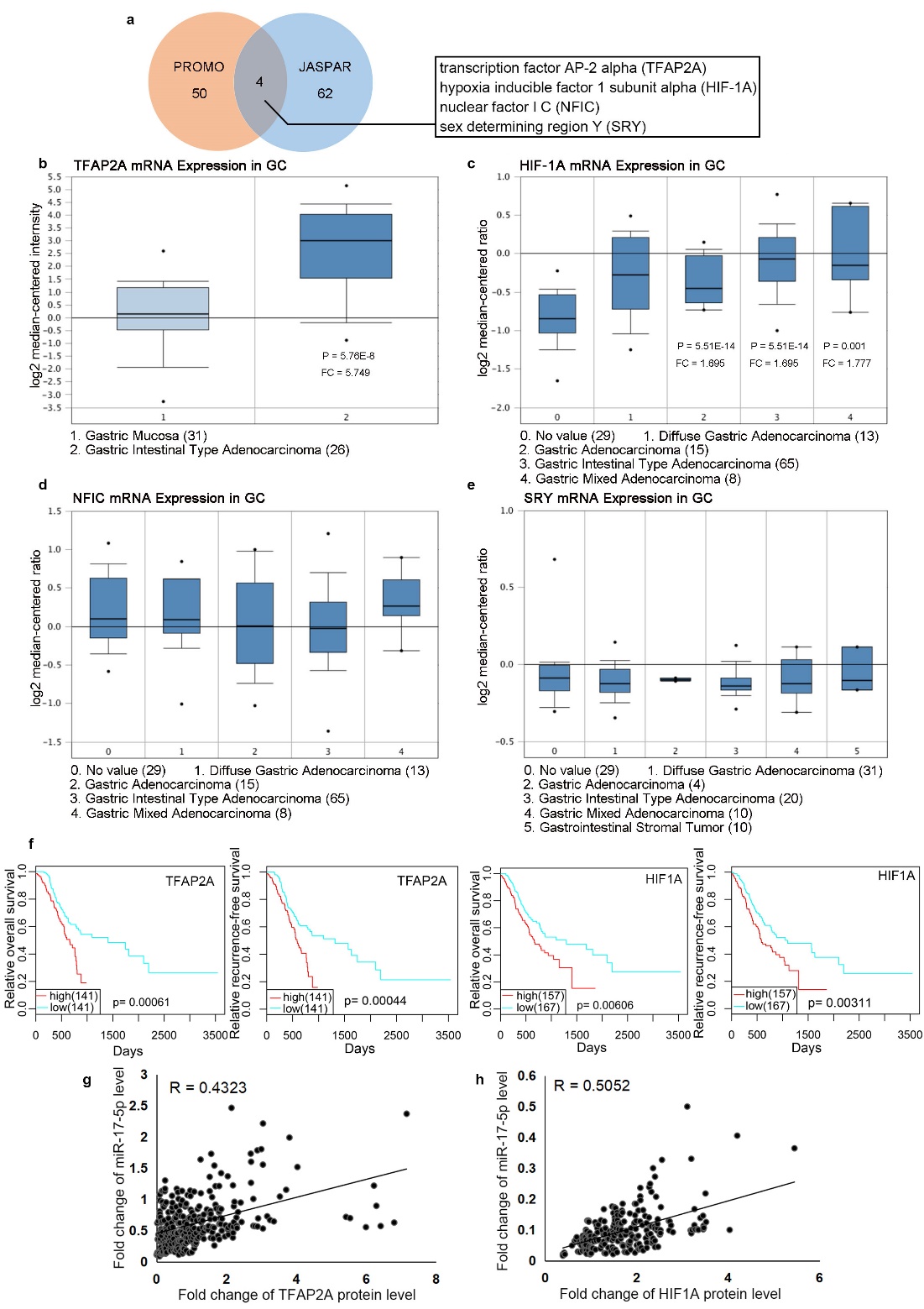


**Figure S5. Analysis of four TFs that potentially activate miR-17-5p transcription.** (a) JASPA and PROMO prediction as well as the Venn diagram for screening putative TFs. (b-e) Analysis of the mRNA levels of four TFs (TFAP2A, HIF-1A, NFIC and SRY) in GC using the Oncomine database. (f) High TFAP2A and HIF-1A expressions were predicted to lead to worse OS and RFS of GC patients (cutoff value: 50% of TFAP2A or HIF-1A mRNA expression). (g and h) miR-17-5p levels were predicted to be positively correlated with high TFAP2A and HIF-1A expressions in GC patients. * P < 0.05.

**Figure. S6.**


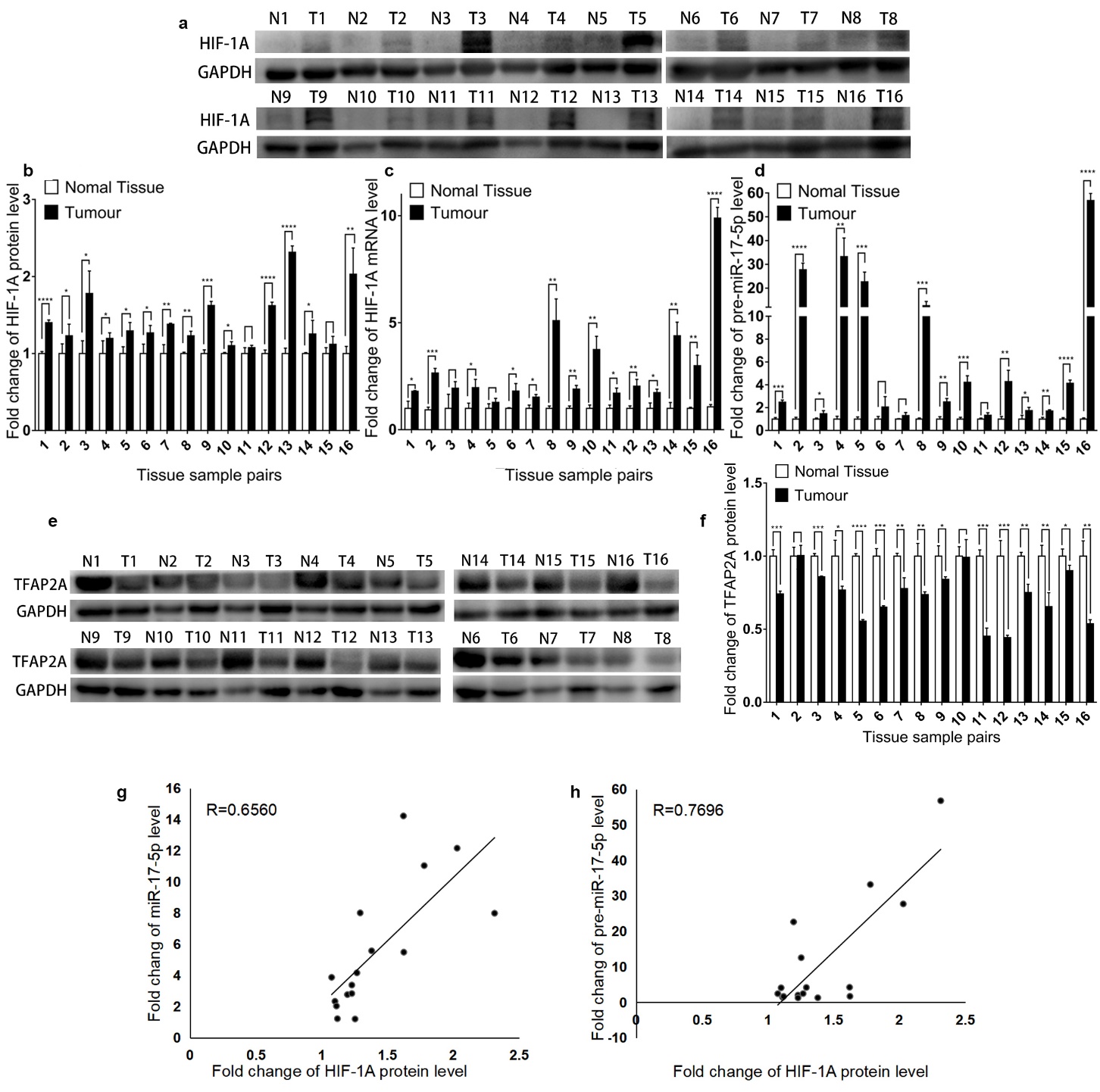


**Figure S6. HIF-1A, pre-miR-17-5p and TFAP2A levels in GC tissue pairs.** (a-c) WB and qRT-PCR analysis: HIF-1A protein and mRNA levels were highly expressed in GC tumor tissues. (d) qRT-PCR analysis: pre-miR-17-5p was upregulated in GC tumor tissues. (e and f) WB and qRT-PCR analysis: TFAP2A protein and mRNA levels were downregulated in GC tumor tissues. (g and h) Pearson’s correlation coefficient: The levels of miR-17-5p and pre-miR-17-5p were positively related to the HIF-1A protein levels in 16 pairs of GC tumor tissues. * P < 0.05; ** P < 0.01; *** P < 0.001; **** P<0.0001.

Figure. S7.


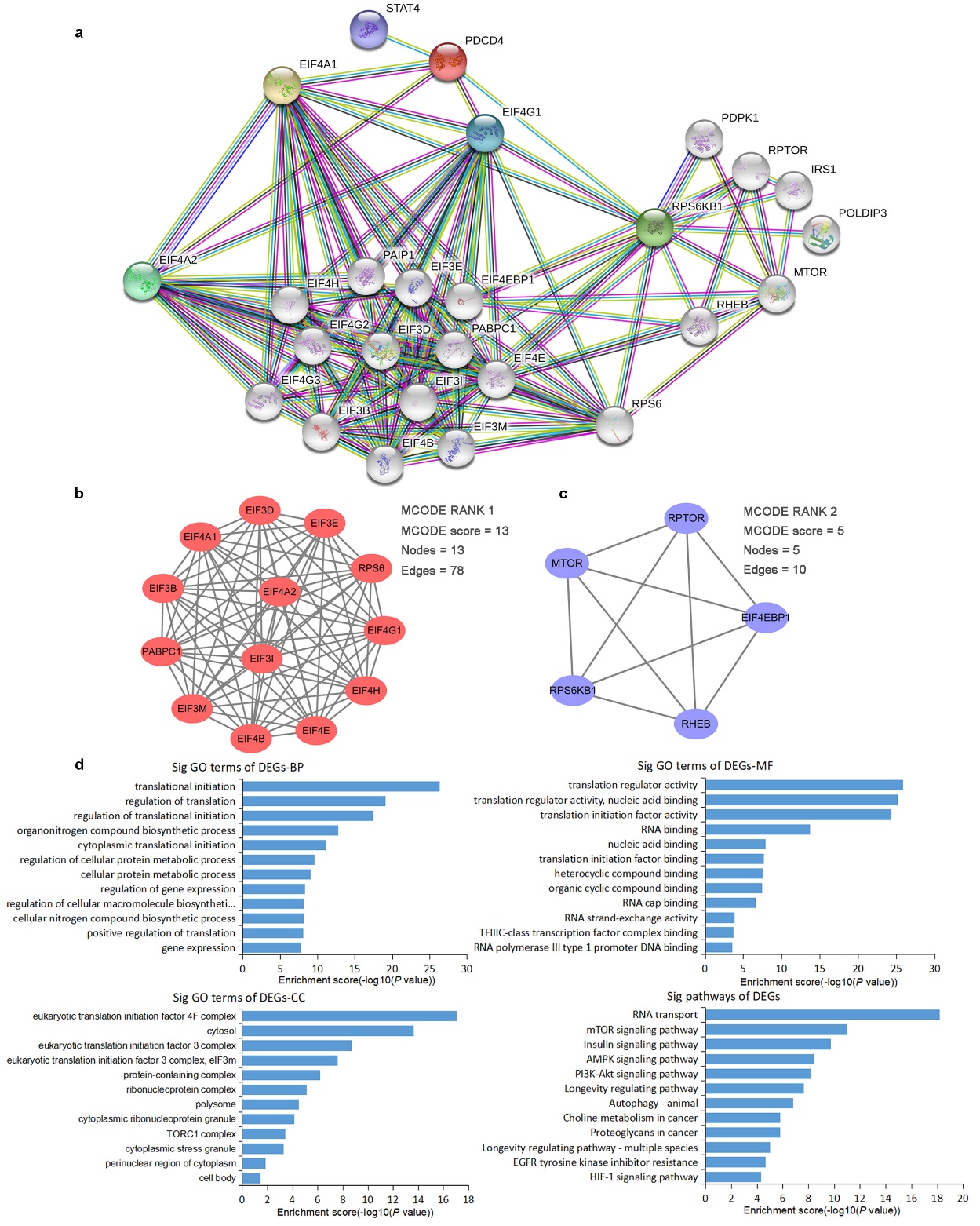


**Figure S7. The PPI network of PDCD4 along with the modules.** (a) Overall PPI network of the genes most connected with PDCD4. (b) Module with MCODE score 13 and nodes 13. (c) Module with MCODE score 5 and nodes 5. (d) Significant GO terms of biological process (BP), molecular function (MF) and cellular component (CC) along with the pathway enrichment analysis of the most connected genes.

Table S1.

**Supplemental Table 1. Clinical features of gastric cancer patients.**

| Case number | Age | Gender | Pathological Stage | Tumour Subtype |
| --- | --- | --- | --- | --- |
| 1 | 63 | M | ⅠB (T2, N0, CM0) | Adenocarcinoma |
| 2 | 65 | M | ⅡB (T2, N2, CM0) | Adenocarcinoma |
| 3 | 64 | M | ⅢA (T3, N2, CM0) | Adenocarcinoma |
| 4 | 70 | F | ⅡA (T3, N0, CM0) | Adenocarcinoma |
| 5 | 62 | M | ⅢB (T3, N3, CM0) | Adenocarcinoma |
| 6 | 50 | F | ⅢB (T3, N3a, CM0) | Adenocarcinoma |
| 7 | 66 | F | ⅢC (T4, N3, CM0) | Adenocarcinoma |
| 8 | 63 | M | ⅢA (T3, N2, CM0) | Adenocarcinoma |
| 9 | 61 | M | ⅢA (T3, N2, CM0) | Adenocarcinoma |
| 10 | 56 | M | ⅡB (T4a, N0, CM0) | Adenocarcinoma |
| 11 | 50 | F | ⅢC (T4a, N2, CM0) | Adenocarcinoma |
| 12 | 56 | M | ⅢC (T4b, N2, CM0) | Adenocarcinoma |
| 13 | 50 | M | ⅢC (T3, N3, CM0) | Adenocarcinoma |
| 14 | 66 | F | ⅢC (T4, N3, CM0) | Adenocarcinoma |
| 15 | 52 | M | ⅢC (T4, N3b, CM0) | Adenocarcinoma |
| 16 | 49 | M | ⅢC (T4a, N3a, CM0) | Adenocarcinoma |

**Table S2.**

**Supplemental Table 2. Sequences of siRNAs and primers.**

| **Item** | **Sequence** |
| --- | --- |
| Control siRNA | UUCUCCGAACGUGUCACGUTT |
| si-PDCD4 | GCGGAAAUGUUAAGAGAUU |
| si-HIF-1A | GCCGAGGAAGAACUAUGAATT |
| GAPDH qRT-PCR primers | Forward: CGAGCCACATCGCTCAGACA |
|  | Reverse: GTGGTGAAGACGCCAGTGGA |
| PDCD4 qRT-PCR primers | Forward: GTTGGCAGTATCCTTAGCATTGG |
|  | Reverse: TCCACATCAGTTGTGCTCATTAC |
| HIF-1AqRT-PCR primers | Forward: TGCATCTCCATCTCCTACCC |
|  | Reverse: CCTTTTCCTGCTCTGTTTGG |
| pre-miR-17-5p qRT-PCR primers | Forward: CAGTGAAGGCACTTGTAGCATTAT |
|  | Reverse: AGTGCAGGGTCCGAGGTATT |
| HIF-1A binding site ChIP primers | Forward: TGATCGCACAGCGTACAC |
|  | Reverse: CAAAGTACTTCCGACCTCCC |
